# miR-3195 suppresses the malignant progression of osteosarcoma cells via targeting SOX4

**DOI:** 10.1186/s13018-023-04321-3

**Published:** 2023-10-30

**Authors:** Jianwei Liang, Dandan Bao, Zhan Ye, Binhao Cao, Guojun Jin, Zhenyu Lu, Jianjun Chen

**Affiliations:** 1 Department of Orthopedics, The First People’s Hospital of Taizhou, No.218 Hengjie Road, Huangyan District, Taizhou City, 318020 Zhejiang Province China; 2 Department of Pharmacy, The First People’s Hospital of Taizhou, No.218 Hengjie Road, Huangyan District, Taizhou City, 318020 Zhejiang Province China

**Keywords:** miR-3195, SRY-box transcription factor 4, Osteosarcoma, Proliferation, Migration, Invasiveness, Apoptosis

## Abstract

**Background:**

Osteosarcoma (OS) is a highly invasive primary malignancy of the bone that is common in children and adolescents. MicroRNAs (miRNAs) are novel diagnostic and predictive biomarkers for cancers. The miRNA miR-3195 is aberrantly expressed in multiple types of tumors. However, the expression levels and biological functions of miR-3195 in OS remain unclear.

**Methods:**

Two Gene Expression Omnibus (GEO) datasets (GSE69470 and GSE16088) were used to analyze differentially expressed miRNAs and mRNAs in osteosarcoma cell lines and OS tissues. Quantitative RT-PCR was used to detect the expression levels of miR-3195 and the SRY-box transcription factor 4 (SOX4) mRNA in OS tissues and cell lines. The relationship between miR-3195 and the 3’-upstream region (3’-UTR) in the SOX4 mRNA (predicted through bioinformatics) was analyzed using Pearson's correlation analysis and confirmed by a dual-luciferase reporter gene experiment. Cell counting kit-8 assays, colony formation assays, flow cytometry, wound healing assays, transwell assays, and western blotting were performed to explore the effects of miR-3195 levels on SOX4 affected OS cell biological behavior.

**Results:**

Our results revealed that miR-3195 was the most down-regulated miRNA and SOX4 was the most up-regulated mRNA by Bioinformatic analysis. It was further confirmed miR-3195 had low expression, and SOX4 had high expression levels in clinical OS tissue samples; the expression levels of both genes were negatively correlated with each other in OS tissues. Overexpression of miR-3195 in OS cell lines significantly inhibited cell proliferation, migration, and invasiveness, while promoting apoptosis; all these effects were reversed by increasing SOX4 expression levels. We also found that miR-3195 could directly bind with the SOX4 gene and down-regulate SOX4 expression.

**Conclusions:**

miR-3195 can modulate proliferation, migration, invasiveness, and apoptosis in OS cells by regulating the SOX4 gene. Thus, the miR-3195/SOX4 signaling may be a novel therapeutic target in OS treatment.

## Introduction

Osteosarcoma (OS) is a highly invasive primary malignancy of the bone that is common in children and young adults; this cancer is also prone to recurrence after therapy [1]. Although great strides have been made over the last few decades in treating OS with primary chemotherapy, tumor excision, and multi-agent adjuvant chemotherapy, patients with recurrent tumors and distant metastases have low survival rates; this is mainly because OS cells develop resistance to chemotherapy. [2] Therefore, there is an urgent need to identify molecular markers associated with malignancy, tumor progression, and resistance to chemotherapy in OS; such markers could also help in identifying potential therapeutic targets for the treatment of OS.

Noncoding RNAs (ncRNAs) are RNA transcripts that are not translated and therefore, do not code for proteins. One class of small endogenous ncRNAs are microRNAs (miRNAs) that are single-stranded, consist of approximately 18 to 24 nucleotides, and are involved in diverse biological processes such as differentiation, growth, invasiveness, metastasis, and musculoskeletal conditions [3–6]. Most miRNAs negatively regulate their target genes by binding to the 3’-untranslated regions (3’-UTRs) of the mRNAs of target genes; this leads to translational silencing of the target gene by destabilizing the target mRNAs, which suppresses protein production [7]. Numerous studies have identified changes in miRNA expression to be associated with the pathogenesis of many diseases, including cancer development and progression [8–10]. The miR-139, [11] miR-361-3p, [12] miR-19, [13] and miR-487a, [14] are known to inhibit the progression of OS by targeting Rho-associated kinases 1 (ROCK1), AT-rich interaction domain 3A (ARID3A), suppressor of cytokine signaling 6 (SOCS6), and B-cell translocation gene 2 (BTG2), respectively. The miR-3195 is over-expressed in castration-resistant prostate cancer, [15] and under-expressed in non-small cell lung cancer [16]. However, none of study has reported about the association between miR-3195 and OS until now.

SRY-box transcription factor 4 (SOX4), SOX4 is a highly conserved transcription factor reported to be associated with a variety of biological processes, including embryogenesis, neural development, and differentiation [17]. Studies have also shown that SOX4 plays a crucial role in the tumorigenesis of several cancers such as triple-negative breast, [18] colorectal, [19] liver cancers, [20] and prostate cancers [21]. According to the study by Chen et al., [22] SOX4 is highly expressed in osteosarcoma tissues and is associated with poor prognosis, knockdown of the SOX4 gene inhibited cell proliferation, migration, invasion, and induced apoptosis in the osteosarcoma cell lines, indicating that it may play an oncogene role in the malignant process of osteosarcoma. To date, limited research has been focused on the mechanisms of miR-3195 and SOX4 with respect to osteosarcoma.

In the present study, we aim to investigate the expression level of miR-3195 in OS tissues and cell lines, and the biological functions and underlying mechanism of miR-3195 in OS cells.

## Materials and methods

### Specimen collection

OS tissues and its paired adjacent healthy soft tissues (distance from cancerous tissue ≥ 5 cm) were obtained from 34 patients with OS (age range, 9–52 years; mean age, 23.72 ± 10.42 years; 13 male patients and 21 female patients) who were admitted to the First People’s Hospital of Taizhou from June 2018 to January 2022. All clinical tissue specimens used in our study were collected, numbered, registered, and immediately frozen in liquid nitrogen and confirmed as OS by a pathologist. The patients included in this study had not received any previous chemotherapy, radiotherapy, immunotherapy or systemic treatment for their disease. Patients or their legal guardians in accordance with the guidelines of in the Declaration of Helsinki. All experiments involving human specimens were approved by the Ethics Committee of the First People’s Hospital of Taizhou (approval no.AF/SC-07/v2.0). All the adult patients or parents of minor patients provided written informed consent prior to specimen acquisition.

### Bioinformatic analysis

Two microarray dataset GSE69470 and GSE16088 were downloaded from Gene Expression Omnibus (GEO) database (https://www.ncbi.nlm.nih.gov/). The miRNA dataset of GSE69470 includes 1 human normal osteoblast cell line (NHOst) and 10 OS cell lines (HOS, Hu09, OHS, SAOS-2, SJSA-1, U-2 OS, KHOS NP, CHA-59, KHOS-240S and KHOS-312H), and the mRNA dataset of GSE16088 includes 14 OS tissues and 6 normal tissues. Differential expressed miRNAs and mRNAs on the retrieved data were analysed using GEO2R (https://www.ncbi.nlm.nih.gov/geo/geo2r/) with the criteria of adjusted *p* < 0.05 and |log2 fold change (FC)|> 2. Volcano map, Potential targets of miR-3195 were searched on TargetScan (http://www.targetscan.org/vert_72/ ) databases, and candidate targets were identified from the predicted results and the differentially up-regulated mRNAs in GSE16088.

### Cell culture

The human OS cell lines including HOS, U2OS, Saos-2, and MG-63 and the normal human osteoblast cell line hFOB1.19 were obtained from the American Type Culture Collection (ATCC, Manassas, VA, USA). OS cell lines were cultured in Roswell Park Memorial Institute (RPMI)-1640 medium (Gibco, Waltham, MA, USA) and hFOB1.19 was cultured in Dulbecco’s modified Eagle’s medium (DMEM) (Gibco, Waltham, MA, USA) containing 2.5 mM L-glutamine (Gibco, Thermo Fisher Scientific, Waltham, MA, USA) and 10% Fetal bovine serum (FBS) (Sigma-Aldrich, St Louis, MO, USA) with 100 U/ml penicillin/streptomycin. All cell lines were cultured in an incubator with a humidified atmosphere at 37 °C and 5% CO_2_, except for hFOB1.19 maintained at 33.5 °C.

### Cell transfection

The miR-3195 mimics and negative controls (NCs) were synthesized by Guangzhou Ribo Bio Co., Ltd. The recombinant human SOX4 overexpression plasmid (pcDNA3.1-SOX4) and empty plasmids (pcDNA3.1) were constructed by GenePharma (Shanghai, China). Logarithmic-phase U2OS and MG-63 cells were seeded at 1 × 10^6^ cells/well into 6-well plates and transfected with the oligonucleotides and plasmids at ∼80% confluency using Lipofectamine 3000 (Invitrogen, Carlsbad, CA, USA) according to the manufacturer's instructions.

### Cell viability assay

The effects of miR-3195 or SOX4 plasmid on cell proliferation were evaluated by the Cell Counting Kit-8 assay (CCK-8) (Beyotime, Shanghai, China). The 100 µL/well (5 × 10^3^) cell suspensions were inoculated in 96-well, incubated at 37 °C with 5% CO_2_ for different points in time. 10 µL of CCK-8 reagent was added to each well and the plates incubated for approximately 2 h. Then, the optical density (OD) was measured at 490 nm using a microplate reader (Victor Nivo 3F, Perkin Elmer), and cell proliferation rate was calculated by OD_490_ value.

### qRT-PCR analysis

The tissues/cells were lysed with Trizol (Invitrogen Life Technologies, Carlsbad, CA, USA) according to the manufacturer's instructions. The extracted RNA was quantified using a NanoDrop 2000 (Thermo Fisher, Waltham, MA, USA). Reverse transcription was carried out using 2 µg of RNA using the One Step PrimeScript® miRNA cDNA Synthesis Kit and PrimeScript® RT Master Mix (Takara, Kusatsu, Shiga, Japan), respectively. Following this, qRT-PCR was performed using the SYBR® Premix Ex Taq™ (Takara, Kusatsu, Shiga, Japan) in a CFX96 Touch instrument (Bio-Rad, Hercules, CA, USA). The sequences of the primers used are:

U6 forward primer: 5’-TTATGGGTCCTAGCCTGAC-3’,

U6 reverse primer: 5’-CACTATTGCGGGTCTGC-3’;

SOX4 forward primer: 5’-CCGAGCTGGTGCAAGACC-3’,

SOX4 reverse primer: 5’-CCACACCATGAAGGCGTTC-3′;

GAPDH forward primer: 5’-TGAAGGTCGGAGTCAACGG-3’,

GAPDH reverse primer: 5’-TCCTGGAAGATGGTGATGGGA-3’;

miR-3195 forward primer: 5’-TGCAAGCGCGCCGGGCCCGGGT-3’,

miR-3195 reverse primer: 5’-GTGCAGGGTCCGAGGT-3’.

The U6 and GAPDH genes were used as endogenous controls for miRNA and mRNA expression levels. Relative quantification analyses were performed using the comparative threshold cycle (CT) method and calculated using the 2^−ΔΔCt^ method.

### Annexin V-FITC/PI analysis

Transfected U2OS and MG-63 cells about 1 × 10^6^ were harvested and washed twice in phosphate buffered saline (PBS) and re-suspended in 500 μL of binding buffer with Annexin V-FITC and PI provided in the Annexin V-FITC Apoptosis Detection kit (BD Biosciences, San Jose, MA, USA) as per the manufacturer's instruction. The cells were incubated at room temperature in the dark for 15 min. Following this, each sample was quantitatively analyzed by flow cytometry using the FACS Calibur flow cytometer (FACSAria, Becton Dickinson).

### Wound healing and cell migration assays

Transfected U2OS and MG-63 cells about 1 × 10^6^ were seeded in six-well plates and allowed to reach confluence in complete media, respectively. The cells were incubated overnight in serum-free medium, and a sterilized 200 μL pipette tip was used to scratch the cell monolayer to create a ‘wound’. The cells were then rinsed with PBS and cultured in complete RPMI-1640. The migration of cells towards the scratch and ‘wound’ closure was observed at 0 and 24 h and photographed under a microscope (Leica, DMI8) from five randomly selected fields in each sample; the wound areas were calculated using the Image J software. For cell migration assays, the membranes of the transwell chambers were pre-coated with Matrigel (BD Biosciences, New Jersey, USA) and each group of transfected cells about 1 × 10^5^ in serum-free medium was added to the upper chamber in 24 well cell culture plate (Corning, Inc, New York, NY, USA) with 8 µm pores. Medium containing 10% FBS was added to the lower chamber and the entire setup was incubated at 37 °C in a humidified incubator containing 5% CO_2_ as per the manufacturer's protocols. After 24 h of culturing, the cells that moved to the lower chamber were stained with 0.1% crystal violet and counted from five different areas under an inverted microscope.

### Western blot analysis

The Western blot assay was performed according to the steps described previously [23, 24]. In brief, transfected cells were harvested and total protein was isolated using RIPA (Beyotime, Shanghai, China) lysis buffer with phenylmethylsulfonyl fluoride (PMSF) (Beyotime, Shanghai, China). The cell homogenates were centrifuged at 20 000 g at 4 °C for 20 min, and the supernatant was collected. The protein concentrations of the samples were determined using the bicinchoninic acid (BCA) (Beyotime, Shanghai, China) assay. Following 12% sodium dodecyl sulfate polyacrylamide gel electropheresis (SDS-PAGE), the proteins were transferred to a PVDF membrane. After blocking with 5% bovine serum albumin (BSA) (Boster Wuhan, China), the PVDF membrane (Millipore, Bedford, MA, USA) was incubated overnight with the primary antibodies: anti-Bax (diluted 1:1000, #5023, Cell Signaling Technology), anti-Bcl-2 (1:1000, #15,071, Cell Signaling Technology), anti-cleaved caspase-3 (1:1000, #9661, Cell Signaling Technology), anti-GAPDH (1:1000, #92,310, Cell Signaling Technology), and anti-SOX4 (1:1000, ab243739, Abcam). Subsequently, the membranes were washed in tris-buffered saline with 0.1% Tween (TBST) and then incubated with the appropriate anti-mouse/rabbit secondary antibody (1:10,000, Abcam). The protein bands were visualized by chemiluminescence and quantified by ChemiDoc Touch (Bio-Rad, Hercules, CA, USA).

### Luciferase reporter assay

Wild-type (wt)- and mutant (mut)-SOX4 3’UTR dual-luciferase reporter plasmids were constructed using the psiCHECK2 reporter vector. The U2OS and MG-63 cells were seeded and cultured in six-well plates overnight, following which, the cells were transfected with the SOX4-wt or SOX4-mut plasmids, and/or the miR-3195 mimic, or NCs. After 48 h, the luciferase activity in the cells was measured using the Dual-Luciferase Reporter Assay System (Promega, Madison, WI, USA). All the luciferase reporter assays were replicated independently three times. The relative luciferase activities were expressed as the ratios of firefly luciferase activities to renilla luciferase activities.

### Statistical analysis

Statistical calculations and drawing were performed using SPSS 22.0 (SPSS Inc., Chicago, IL, USA) and GraphPad Prism 6 (GraphPad Software Inc., San Diego, CA, USA). Data are presented as the mean ± standard deviation (SD). Student's t-test was used for comparisons between two groups, and one-way or two-way with post hoc analysis of variance was used for comparisons among multiple groups. Differences with* P* < 0.05 were considered as statistically significant.

## Results

### The expression of miR-3195 was down-regulated in OS tissues and cell lines

**Fig. 1:**
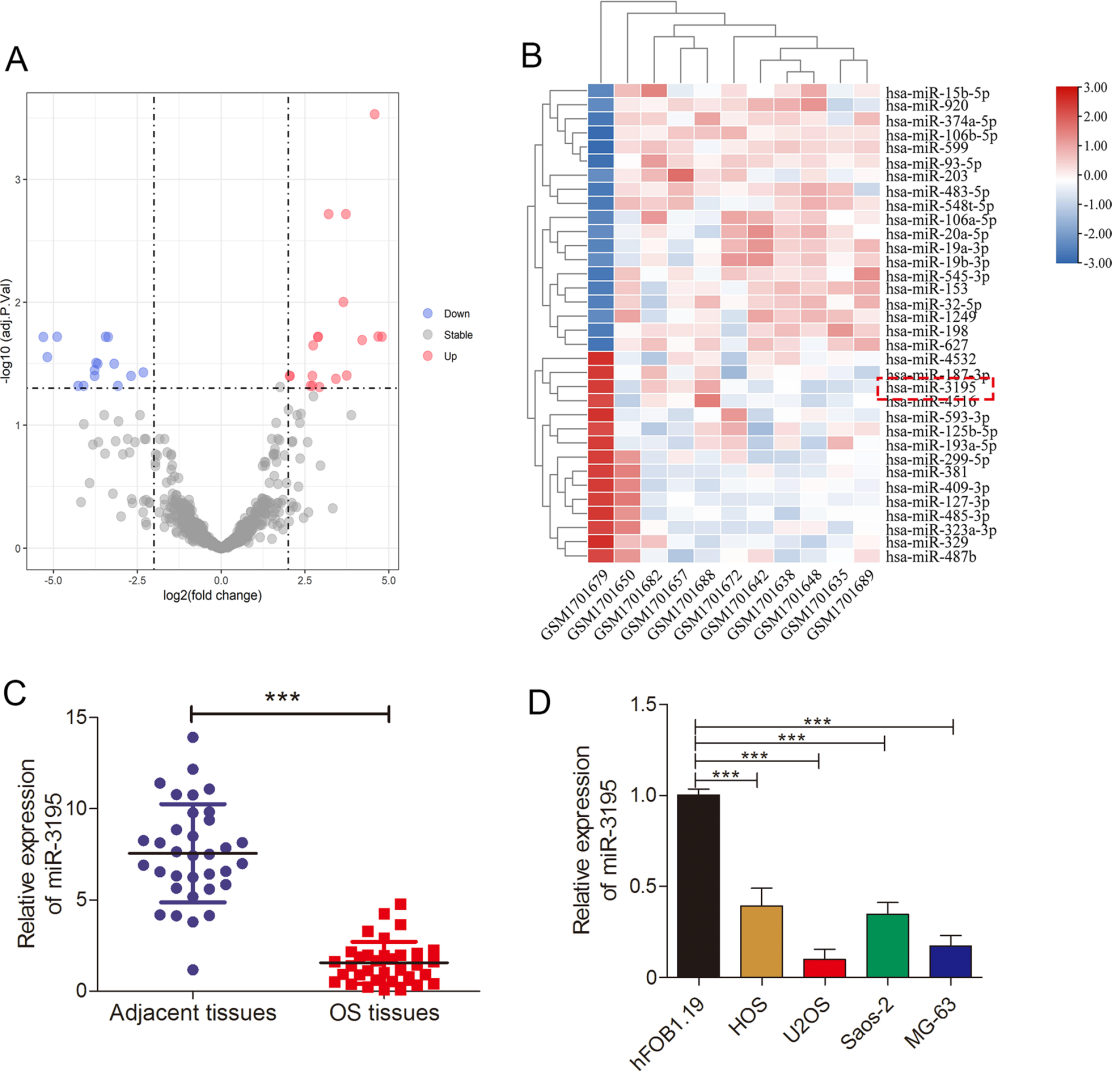
miR-3195 was down-regulated in OS tissues and cell lines. **A** The volcano plot screen differentially expressed miRNAs in GSE69470 database. **B** Heatmap show the different expression of miRNAs. **C** The miR-3195 expression in OS tissues and matched normal adjacent tissues was measured by quantitative real-time PCR. **D** The expression level of miR-3195 in OS cell lines (HOS, U2OS, MG-63, and Saos-2) and osteoblastic cell lines hFOB1.19 measured by quantitative real-time PCR. Data are presented as mean ± standard deviation (SD) of three independent experiments. ***indicates *P* < 0.001

To investigate the function of miR-3195 in OS, we first analyzed data on the expression levels of miR-3195 from publicly available databases, clinical samples, and cell lines. A total of 34 differentially expressed miRNAs were obtained in GSE69470 from the GEO database after background correction and normalization. In the GSE69470 database, 19 miRNAs were over-expressed, and 15 were under- expressed (Fig. 1A); of these, miR-3195 was found to be down-regulated (Fig. 1B). To verify these results, we measured miR-3195 expression levels in OS tissues using qRT-PCR and found that the levels of miR-3195 were significantly lower in OS tissues than in the adjacent normal tissues (Fig. 1C). We also evaluated miR-3195 expression levels in the OS cell lines HOS, U2OS, MG-63, and Saos-2 and osteoblastic cell lines hFOB1.19. The expression levels of miR-3195 in the HOS, U2OS, MG-63, and Saos-2 cell lines were significantly lower than those in the hFOB1.19 cell line (Fig. 1D). Collectively, these findings confirm that miR-3195 expression is down-regulated in OS tissue and cell lines.

### Over-expression of miR-3195 inhibited the proliferation of OS cell lines and induced apoptosis

**Fig. 2:**
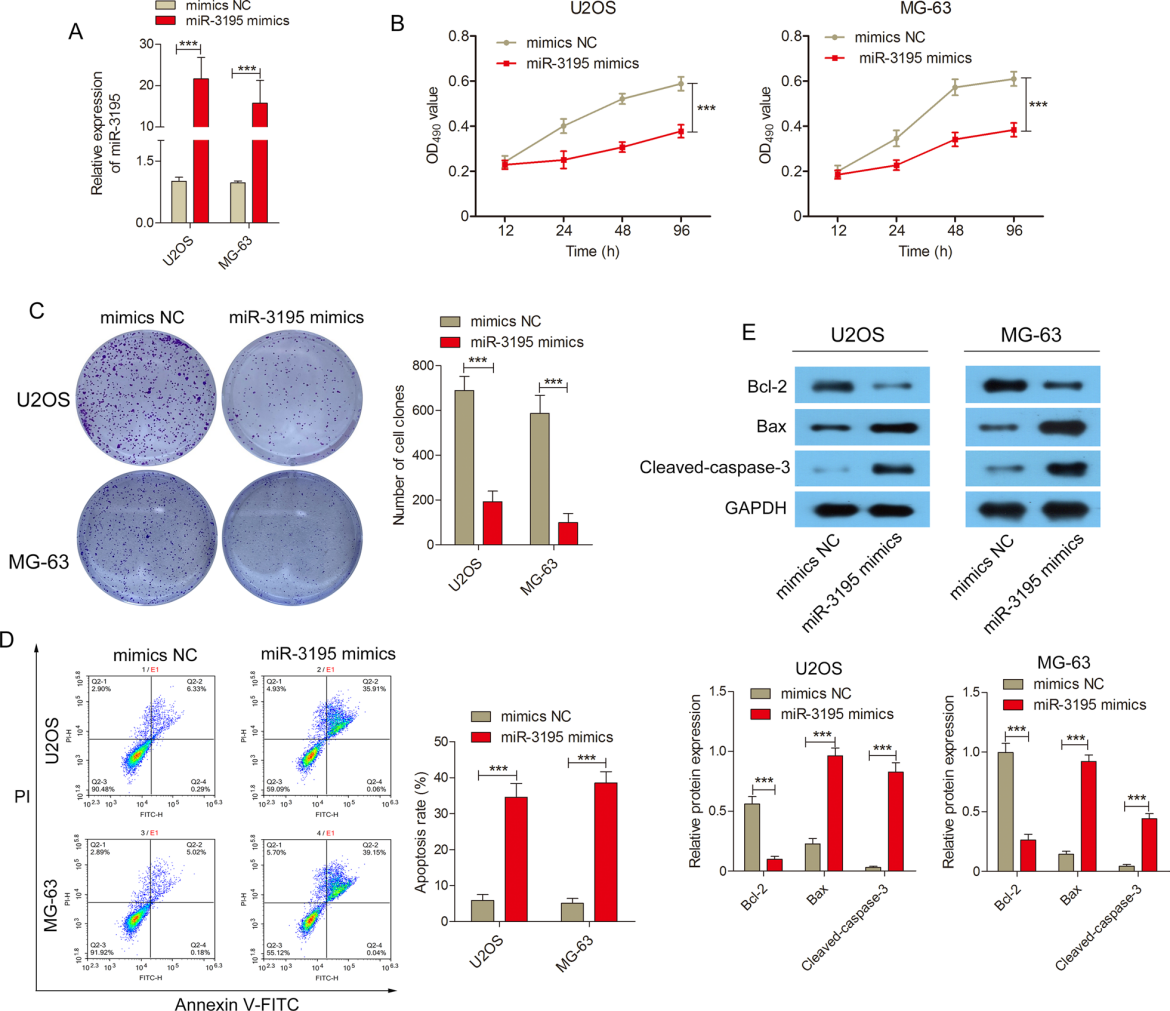
Overexpression of miR-3195 inhibited OS cell proliferation and induced cell apoptosis. **A** The transfection efficiency of miR-3195 mimic or miR-NC transfected with in U2OS and MG-63 cells was detected by qRT-PCR. **B** Cell viability was measured (12—96 h) via CCK-8 assays in transfected U2OS and MG-63 cells. **C** Colony formation ability was measured in transfected U2OS and MG-63 cells by colony formation assay. **D** The level of apoptosis rate in transfected U2OS and MG-63 cells was detected by flow cytometry. **E** The expression levels of apoptosis rated protein Bcl-2, Bax and Cleaved-caspase-3 in transfected U2OS and MG-63 cells were detected by Western blotting. Data are presented as mean ± SD of three independent experiments. ***indicates *P* < 0.001

To investigate the effects of miR-3195 over-expression on OS cells, we transfected U2OS and MG-63 cells with miR-3195 mimics and NCs. A qRT-PCR conducted to evaluate the transfection efficiency found that miR-3195 expression was significantly higher in U2OS and MG-63 cells transfected with miR-3195 mimics as compared to the untransfected cells and those transfected with the NCs (Fig. 2A). The CCK-8 assay revealed that OS cells transfected with miR-3195 mimics had significantly lower proliferation abilities than those transfected with the NCs (Fig. 2B). The colony formation assays showed that transfection with miR-3195 mimics significantly reduced the colony-forming ability of cells, as well as increased apoptosis in these cells as compared to those transfected with the NCs; (Fig. 2C and D). To further confirm the role of miR-3195 over-expression on the increased apoptosis rate, western blotting was used. We found that the levels of the anti-apoptosis protein, Bcl-2, were decreased and that the levels of the pro-apoptosis proteins, cleaved-caspase-3 and Bax, were increased in the cells transfected with miR-3195 mimics as compared to those transfected with the NCs (Fig. 2E). These results suggest that over-expression of miR-3195 inhibited the proliferation and colony formation abilities of OS cells and promoted apoptosis in these cells.

### Over-expression of miR-3195 suppressed the migration capacity and invasiveness of OS cells

**Fig. 3:**
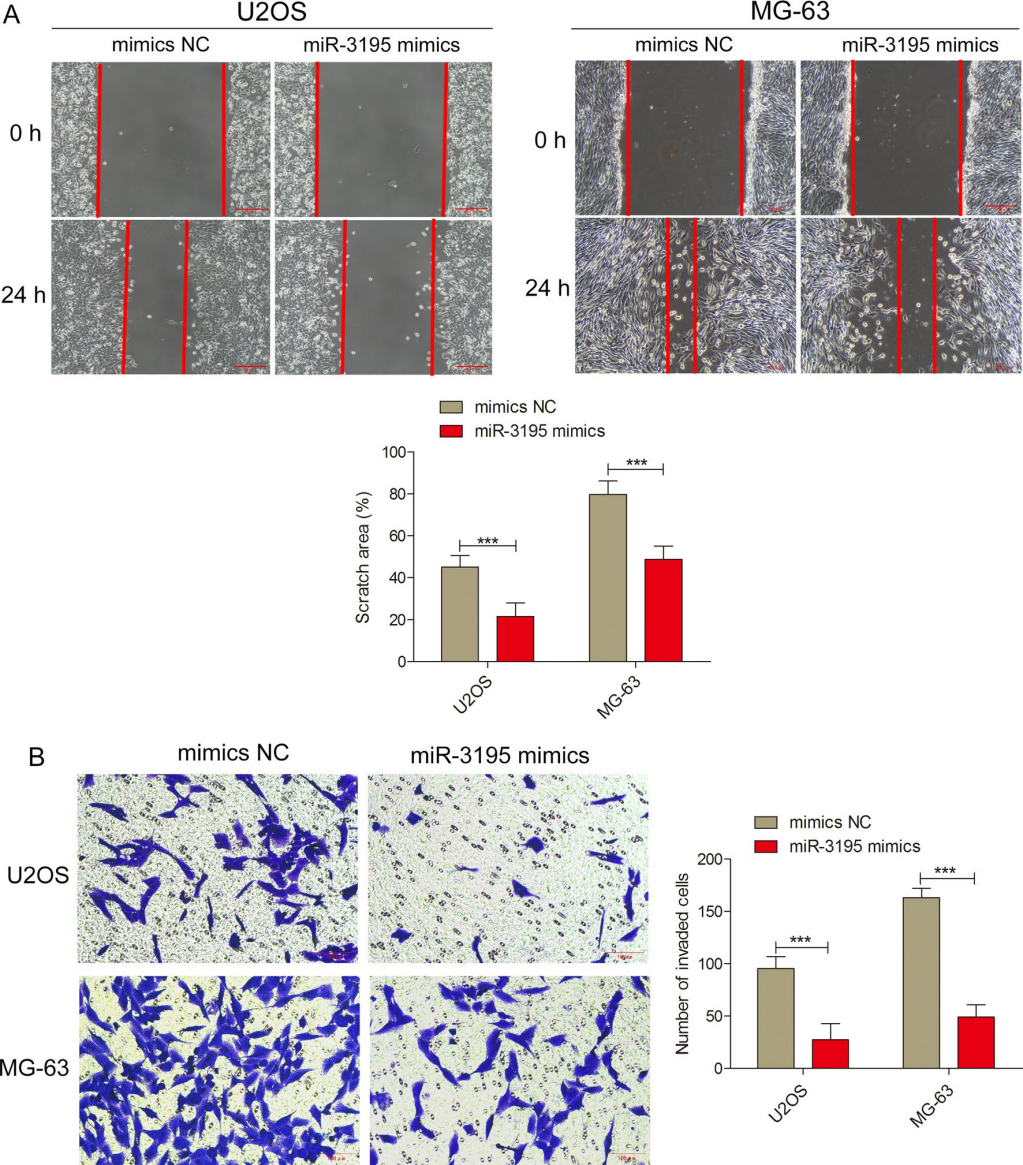
Overexpression of miR-3195 suppressed the migration and invasion capacity of OS cells. **A** After transfection with miR-3195 mimics, miR-NC, OS monolayers were scratched to induce horizontal migration of U2OS and MG-63 cells at 0 h and 24 h. Scale bars are 200 µm; **B** Transwell invasion assay was performed in U2OS and MG-63 cells transfected with miR-3195 mimics, miR-NC. At 24 h after plating, cells that had migrated to the underside of the filters were fixed and stained with crystal violet. Representative images of invasive cells are shown. The number of invasive cells was counted. Scale bars are 100 µm. Data are presented as mean ± SD of three independent experiments. ***indicates *P* < 0.001

To assess the effect of miR-3195 on the mobility of OS cell lines, wound healing assays and transwell assays were performed. The OS cell lines that had been transfected with miR-3195 mimics had significantly lower wound healing abilities than those which had been transfected with the NCs (Fig. 3A). The number of OS cells transfected with miR-3195 mimics that had migrated through the matrigel was significantly lower than those which had been transfected with the NCs (Fig. 3B). These results demonstrate that miR-3195 negatively regulates migration and invasiveness in OS cell lines.

### SOX4 is a direct target of miR-3195

**Fig. 4:**
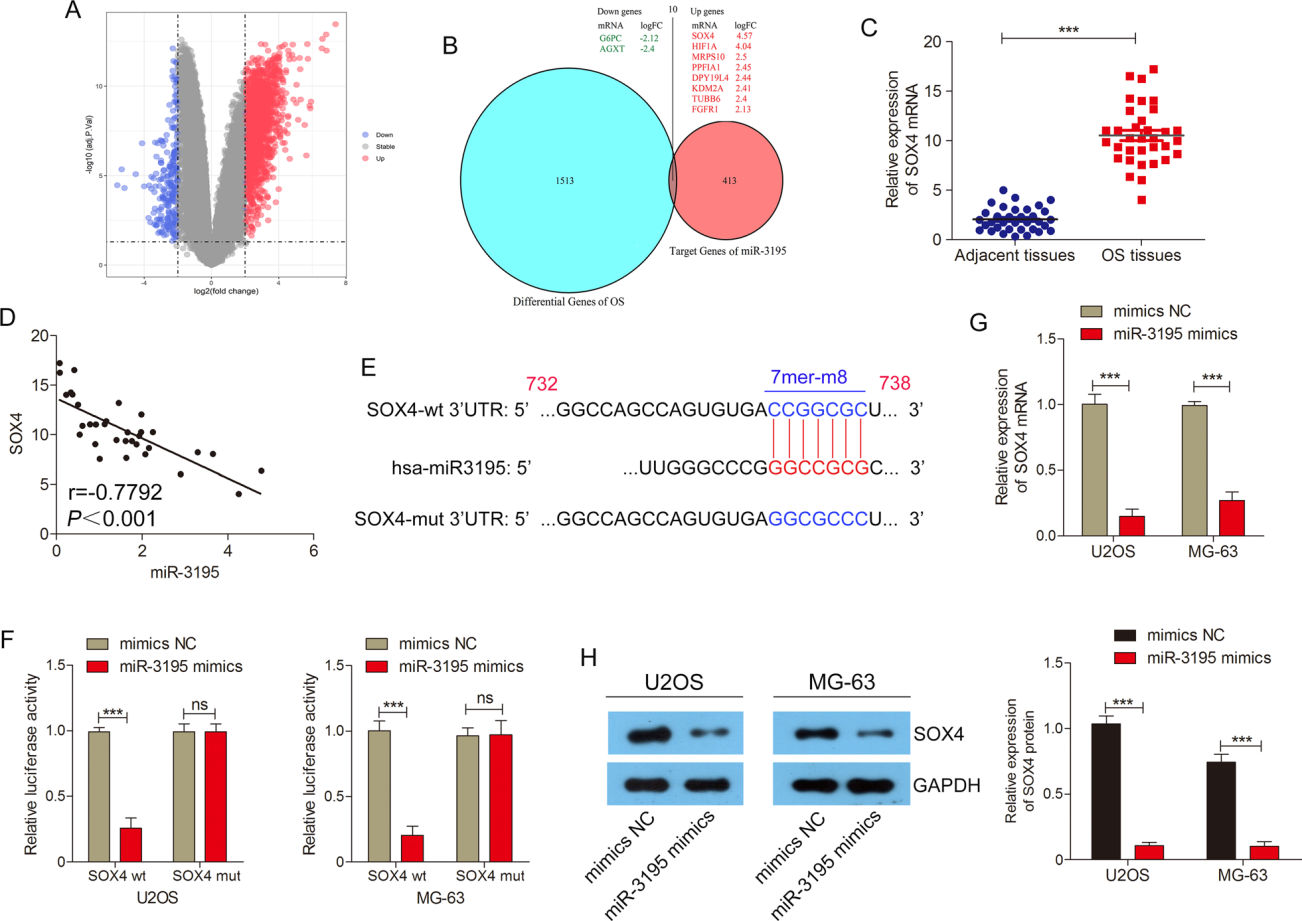
SOX4 is a direct target of miR-3195. **A** The volcano plot screen differentially expressed mRNAs in GSE16088 database. **B** Venn diagram of predicted target genes using TargetScan database and differentially expressed genes in GSE16088. **C** Expression level of SOX4 mRNA in OS tissues and adjacent normal tissues. **D** Correlation analysis between SOX4 and miR-3195 expression levels in OS tissues was analysed by Pearson's correlation analysis. **E** The wild-type (WT) and mutant (mut) 3’-UTR (untranslated region) of SOX4 contained the complementary sequences of miR-3195. **F** Luciferase activity was measured in MG-63 cells co-transfected with miR-3195 mimics or mimics NC and wild type (wt) or mutant (mut) 3ʹUTR of SOX4. (G-H) Relative expression of SOX4 mRNA and protein after overexpressing miR-3195 were assessed by qRT-PCR and Western blot, respectively. Data are presented as mean ± SD of three independent experiments. ***indicates *P* < 0.001, ns indicates *P* > 0.05

To further investigate the downstream regulatory mechanism by which miR-3195 affects the proliferation, migration, and invasiveness of OS cells, we combined gene expression analysis with target prediction methods to identify the genes targeted by miR-3195. The expression profile data obtained from the GSE16088 dataset (including 14 OS tissue samples and 6 normal tissue samples) were preprocessed and assessed for quality using GEO2R. According to the set cut-off criteria (*P* < 0.05 and |log2FC|> 2.0), a total of 1524 differentially expressed genes (between the OS tissues and normal tissues) were identified; among these, 1322 mRNAs were up-regulated, and 202 mRNAs were down-regulated (Fig. 4A). The TargetScan database, which was used to predict the gene targeted by miR-3195, identified a total of 424 genes. Of these, 10 candidate genes were found to also occur in the pool of differentially expressed genes identified previously (Fig. 4B). Out of these 10 genes, we chose to investigate the significantly up-regulated gene SOX4. In 34 cases, SOX4 was highly expressed and negatively correlated with miR-3195 levels in OS tissues (Fig. 4 C and D). Based on the binding sites in the 3’UTR of SOX4 and miR-3195, and the results of the dual-luciferase reporter assay, we were able to ascertain how these molecules could bind with each other (Fig. 4 E and F). The results of the qPCR and western blot analyses revealed that the levels of SOX4 mRNA and protein were markedly down-regulated in OS cell lines transfected with miR-3195 mimics as compared to those which had been transfected with the NCs (Fig. 4 G and H). All the above results demonstrate that SOX4 is a target of miR-3195.

### Over-expression of SOX4 partially reverses the inhibitory effects of miR-3195 in OS cell lines

**Fig. 5:**
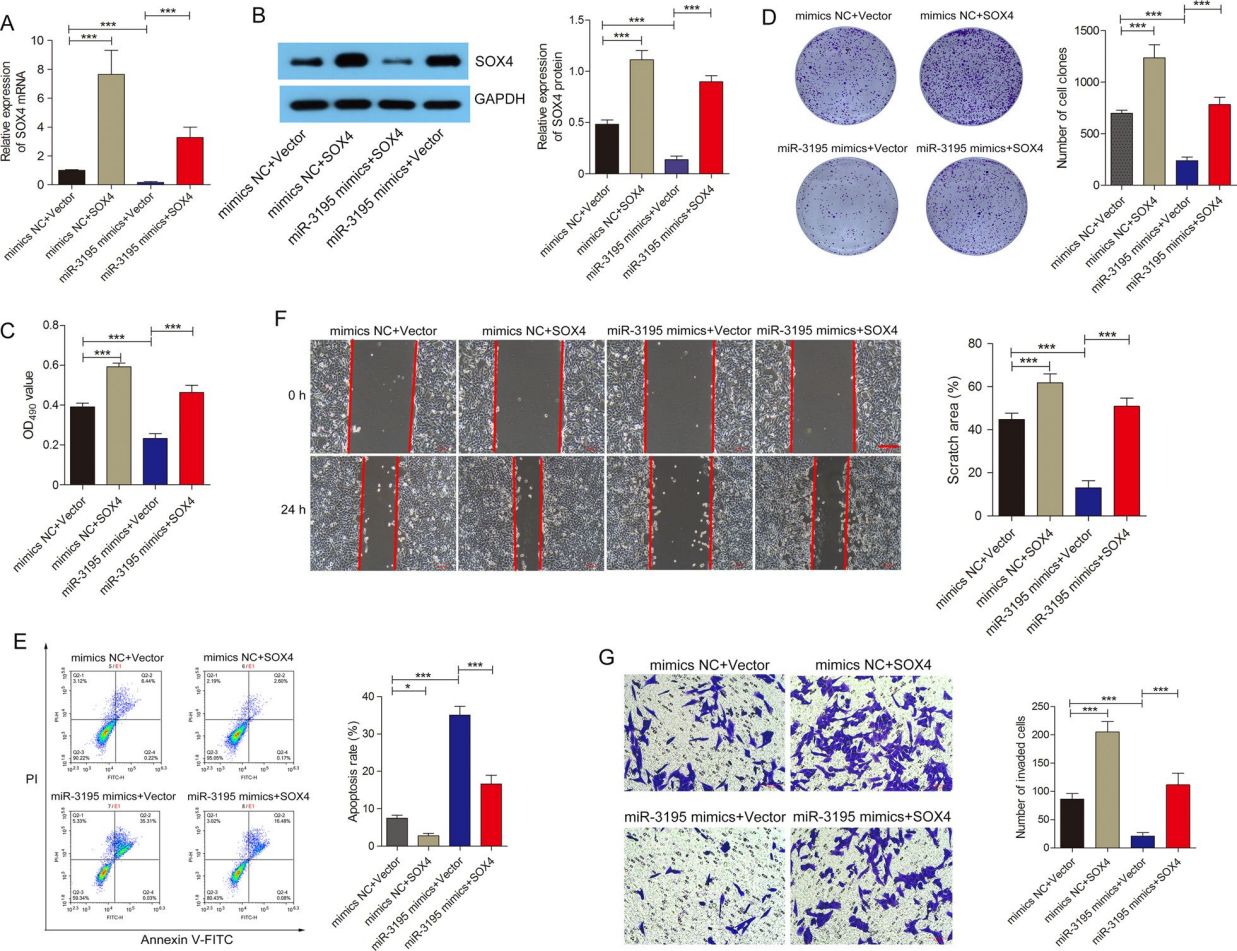
Overexpression of SOX4 partially reverse the inhibitory effects of miR-3195 for OS progression. **A**-**B** Relative expression of SOX4 mRNA and protein after MG-63 cells co-transfected with miR-3195 mimics and SOX4 plasmid. **C** CCK-8 assays was performed to evaluate the cell viability after MG-63 cells co-transfected with miR-3195 mimics and SOX4 plasmid. **D** Colony formation ability was measured in MG-63 cells co-transfected with miR-3195 mimics and SOX4 plasmid. **E** The level of apoptosis rate in co-transfected with miR-3195 mimics and SOX4 plasmid was detected by flow cytometry. **F** Images of measurements of cell migration in vitro scratch assay with MG-63 cells co-transfected with miR-3195 mimics and SOX4 plasmid at 0 h and 24 h. Scale bars are 200 µm. **G** Transwell invasion assay was performed in MG-63 cells co-transfected with miR-3195 mimics and SOX4 plasmid. Scale bars are 100 µm Data are presented as mean ± SD of three independent experiments. *indicates *P* < 0.05, ***indicates* P* < 0.001

To further investigate the role of the miR-3195/SOX4 siganling in OS, we transfected OS cell lines with pcDNA3.1-SOX4 and miR-3195 mimics. We found that the expression of SOX4 was markedly increased in such co-transfections as compared to those which were co-transfected with the NCs and empty vector. The cells co-transfected with miR-3195 mimics and empty vector had lower levels of SOX4 expression than those cells co-transfected with the NCs and empty vector. The cells co-transfected with miR-3195 mimics and pcDNA3.1-SOX4 had higher expression levels of SOX4 than cells co-transfected with miR-3195 mimics and empty vector (Fig. 5A and B). The viabilities and colony formation abilities of the co-transfected cells showed consistent changes as per the expression levels of SOX4 in each group (Fig. 5C and D); the migration abilities and invasiveness of the co-transfected cells showed similar patterns (Fig. 5F and G). We found that the over-expression of SOX4 or miR-3195 could reduce or increase apoptosis in the co-transfected cells as compared to those in cells co-transfected with NCs and empty vector, respectively. Cells co-transfected with miR-3195 mimics and pcDNA3.1- SOX4 had lower apoptosis rates as compared to those co-transfected miR-3195 mimics and empty vector (Fig. 5E). Taken together, these findings suggested that miR-3195 likely targets and inhibits SOX4 expression, which in turn, inhibits the malignant progression of OS.

## Discussion

The discovery of miRNAs in cancer has opened up a new realm in biomarkers and targeted gene therapy for researchers in recent years. The miRNAs can control the expression of target genes by modifying the mRNAs of the target genes; therefore, the over- or under-expression of such miRNAs can down- or up-regulate the expression of the target genes [25]. Hence, miRNAs have been intensely studied as biomarkers that correlate with the progression of various malignancies [26, 27]. In this study, we observed that miR-3195 was under-expressed in human OS tissue and cell lines and that it has the potential to inhibit the malignant progression of OS. Specifically, we demonstrated that the up-regulation of miR-3195 expression could inhibit cancer cell migration and invasiveness, and promote apoptosis in OS cells. Furthermore, we investigated the mechanisms by which miR-3195 affects these processes in OS cell lines using a target gene prediction software and dual-luciferase reporter assay. Our results indicate that SOX4 is a direct target of miR-3195. We have also demonstrated the involvement of SOX4 in the functional consequences of miR-3195 over- expression. Over-expression of miR-3195 inhibited SOX4 expression, resulting in impaired cell proliferation, migration, and invasiveness, and also promoted apoptosis in OS cell lines.

The role of miR-3195 in the onset and progression of several types of cancers has been investigated. For example, the expression of miR-3195 is significantly up-regulated in prostate cancer and has been associated with enhanced PC3 cell migration [15]. However, the expression of miR-3195 is down-regulated in non-small cell lung cancer tissues and cell lines; in this cancer, tissues with low expression levels of miR-3195 showed lymph node metastasis and advanced TNM stages [28]. Similarly, the expression of miR-3195 was low and might act as tumor suppressors in laryngeal cancer [29]. Additionally, a latest study had reported that the miR-3195 was associated with both progression-free survival (PFS) and overall survival (OS) in ovarian clear cell carcinoma [30]. These findings suggest that miR-3195 affects tumor progression in different ways, and may be a useful prognostic marker in these cancers. However, the function of miR-3195, especially in OS was still unclear.

The SOX4 gene is a member of the Sry-related high-mobility group box (Sox) family of transcription factors and is closely related to the development of organs and tissues. It is an oncogene and an essential developmental gene that regulates cell proliferation, migration, invasiveness, apoptosis, and epithelial-to-mesenchymal transition through multiple developmental pathways [17]. Previous studies have reported that the expression of SOX4 is up-regulated in all major cancer types, including lung, bladder, prostate, hepatic, hematopoietic cancers, as well as in OS [20, 31–33]. Other studies have also shown that SOX4 is regulated by miRNAs in OS and positively influences OS progression. For instance, miR-88 suppresses the proliferation, migration and invasiveness of pediatric OS; miR-88 also induced apoptosis in this cancer and exerts these effects by targeting SOX4 [34]. In our study, we have confirmed that miR-3195 served as a tumor suppressor in OS by targeting SOX4. Previous study reported that miR-363-3p inhibited the proliferation, migration, and invasiveness of OS cells by directly targeting SOX4 [35]. Similarly, other studies have shown that miR-212, which is significantly down-regulated in human OS tissues, can inhibit the proliferation and invasiveness of OS cells by down-regulating the expression of SOX4 [36]. However, the mechanism by which miR-3195 expression affects OS cells was still not clear. Here we present a possible mechanism by which miR-3195 negatively affects the OS progression; we show that over-expression of miR-3195 down-regulates SOX4 expression in OS cells. This study is consistent with previous reported that SOX4 expression was high in OS tissues [35, 36]. We found that miR-3195 could directly target the 3’-UTR of the SOX4 gene, and suppress SOX4 expression in OS cell lines. Thus, the miR-3195–SOX4 signaling provides a novel therapeutic target for the treatment of OS. In addition, these findings are just in vitro and more experiment should be done to validate in vivo in the future.

This study shows, for the first time, that miR-3195 suppresses OS cell growth, migration, and invasiveness, and also induces apoptosis in these cells by directly targeting SOX4 expression; increased SOX4 expression is known to promote the malignance in OS cells. The newly identified miR-3195/SOX4 axis has shed new light on the miRNA-based regulatory mechanisms involved in cancer progression. Considering the crucial role of miR-3195 in OS, manipulating miR-3195 expression levels may hold potential as a novel therapeutic intervention for treating OS. But, the limitation of this study lies in the utilization of soft tissue as a healthy control tissue for osteosarcoma, due to osteosarcoma is originated from undifferentiated mesenchymal tissue with the ability to directly generate bone-like tissues and bone.

## Data Availability

The data supporting the findings of this study are available from the corresponding author upon reasonable request.

## Abbreviations

OS: Osteosarcoma
ncRNAs: Noncoding RNAs
ROCK1: Rho-associated kinases 1
ARID3A: AT-rich interaction domain 3A
SOCS6: Suppressor of cytokine signaling 6
BTG2: B-cell translocation gene 2
SOX4: SRY-box transcription factor 4
GEO: Gene Expression Omnibus
DMEM: Dulbecco’s modified eagle’s medium
FBS: Fetal bovine serum; NCs: negative controls
CCK-8: Cell counting kit-8
PBS: Phosphate buffered saline
BCA: Bicinchoninic acid
PVDF: Polyvinylidene fluoride
PMSF: Phenylmethylsulfonyl fluoride
TBS: Tris-buffered saline
WT: Wild-type
